# Multiword units lead to errors of commission in children's spontaneous production: “What corpus data can tell us?*”

**DOI:** 10.1111/desc.13125

**Published:** 2021-06-01

**Authors:** Stewart M. McCauley, Colin Bannard, Anna Theakston, Michelle Davis, Thea Cameron‐Faulkner, Ben Ambridge

**Affiliations:** ^1^ Department of Communication Sciences and Disorders University of Iowa Iowa City Iowa USA; ^2^ Department of Psychological Sciences University of Liverpool, Liverpool, Merseyside United Kingdom of Great Britain and Northern Ireland; ^3^ Division of Human Communication, Development & Hearing University of Manchester, Manchester Manchester UK; ^4^ Linguistics and English Language University of Manchester, Manchester Manchester UK

**Keywords:** chunking, corpus analysis, language acquisition, questions

## Abstract

Psycholinguistic research over the past decade has suggested that children's linguistic knowledge includes dedicated representations for frequently‐encountered multiword sequences. Important evidence for this comes from studies of children's production: it has been repeatedly demonstrated that children's rate of speech errors is greater for word sequences that are infrequent and thus unfamiliar to them than for those that are frequent. In this study, we investigate whether children's knowledge of multiword sequences can explain a phenomenon that has long represented a key theoretical fault line in the study of language development: errors of subject‐auxiliary non‐inversion in question production (e.g., “why we can't go outside?*”). In doing so we consider a type of error that has been ignored in discussion of multiword sequences to date. Previous work has focused on errors of omission – an absence of accurate productions for infrequent phrases. However, if children make use of dedicated representations for frequent sequences of words in their productions, we might also expect to see errors of commission – the appearance of frequent phrases in children's speech even when such phrases are not appropriate. Through a series of corpus analyses, we provide the first evidence that the global input frequency of multiword sequences (e.g., “*she is going*” as it appears in declarative utterances) is a valuable predictor of their errorful appearance (e.g., the uninverted question “what she is going to do?*”) in naturalistic speech. This finding, we argue, constitutes powerful evidence that multiword sequences can be represented as linguistic units in their own right.

## 1 INTRODUCTION

Traditionally, language development has been seen as a matter of rapidly abstracting away from concrete linguistic experience and mastering the types of abstract categories and structures long posited under formal linguistic analyses. The resulting knowledge of language is then assumed to consist of separate knowledge of words (e.g., [“man”] [“walk”]), categories (e.g., [NOUN], [VERB]) and rules (e.g., [SUBJECT] [VERB] [OBJECT] word order). The past decade, however, has seen an explosion of psycholinguistic research suggesting that language users remember and actively utilize specific sequences of words taken directly from experience. The frequency of these units—or “chunks”—has been shown to facilitate processing in adult comprehension (e.g., Arnon & Snider, 2010; Bannard, 2006; Reali & Christiansen, 2007) as well as production (e.g., Janssen & Barber, 2012). These findings have received further support from event‐related brain potentials (Tremblay & Baayen, 2010) and eye‐tracking data (Siyanova‐Chanturia et al., 2011).

Psycholinguistic work with children has served to bolster these findings, highlighting a key role for multiword sequences in development (see Theakston & Lieven, 2017 for an overview). For instance, Bannard and Matthews (2008) found that, when controlling for substring (words and word pairs) frequency, overall four‐word sequence frequency predicted the speed and accuracy with which 2‐ and 3‐year‐olds produced compositional phrases. As an example, the high‐frequency sequence “*a lot of noise”* is produced faster and more accurately than the matched, low‐frequency sequence “*a lot of juice.”* Moreover, multiword units exhibit the same type of age‐of‐acquisition effects as do individual words, when age‐of‐acquisition is determined by either subjective ratings or by corpus‐based metrics (Arnon et al., 2017). Taken together, these findings underscore the possibility that multiword chunks serve as building blocks for language learning.

Such findings have played a role in more general theoretical debates over the nature of grammatical development, as highlighted by computational modeling work which has shown that children's early productive speech can be well accounted for by productive grammars which have multiword sequences as a core component (Bannard et al., 2009), and that abstraction over stored sequences can lead to a considerable amount of linguistic productivity (e.g., Solan et al., 2005). Even models lacking abstraction have served to demonstrate that associative learning of chunks from naturalistic input can account for a substantial portion of children's language production (McCauley & Christiansen, 2019a), while subsequent work has shown that computationally straightforward processes of prediction and recognition can give rise to item‐based schemas of the sort postulated in usage‐based theories of development (McCauley & Christiansen, 2019b).

While there is much evidence that children's fluency in producing word sequences can be related to the familiarity of the target phrase, this only represents one of the types of errors that we might expect to result from variation in children's knowledge of different sequences. Another type of error that is known to arise under such circumstances is the error of *commission* or “habit slip” (see e.g., Reason, 1990), whereby a well‐learned behavior occurs even in contexts where it is inappropriate. Evidence that familiar multiword sequences “intrude” inappropriately into children's productions would constitute particularly powerful evidence that children have dedicated representations for such sequences.

**RESEARCH HIGHLIGHTS**

- Recent decades have seen mounting evidence that children are sensitive to the properties (e.g., frequency) of compositional word sequences.
- Previous research has focused on the role of multiword units in protecting against errors of *omission*.
- By analyzing *wh‐* questions appearing in children's spontaneous productions, we find the first evidence that the global input frequency of multiword sequences is a predictor of their errorful appearance, or intrusion into utterances.
- Our finding that multiword units can shape errors of *commission* constitutes particularly powerful evidence that such sequences constitute linguistic units in their own right.

In the present study, we test the possibility that knowledge of multiword sequences might account for errors (of both omission and commission) in *wh‐* questions; one of the few sentence types for which English‐speaking children reliably make word‐order errors (e.g., Estigarribia, 2010; Klima & Bellugi, 1966; Stromswold, 1990), specifically *non‐inversion* (or *uninversion*) errors:

* What they are doing over there ? *
* Why I can't go outside ? *
* Where the biscuits have gone ? *

Traditionally, such errors have been explained in terms of children's failure to master syntactic movement (of the auxiliary to pre‐subject position; e.g., *they are* → *are they*), particularly for adjunct *wh*‐words such as *how* and *why* (e.g., de Villiers, 1991; Stromswold, 1990) and/or auxiliary DO (e.g., Santelmann et al., 2002; Stromswold, 1990). Although some studies have found higher error rates for these types of questions (e.g., Hattori, 2003; Pozzan & Valian, 2017), others have not (e.g., Ambridge et al., 2006; Rowland, 2007; Ambridge & Rowland, 2009).

Evidence suggesting the importance of multiword chunks in children's question formation comes from the studies of Rowland and Pine (2000), Dabrowska (2001), Rowland (2007), Dabrowska and Lieven (2005) and Ambridge and Rowland (2009). All of these studies found some link between the occurrence of particular question types in children's input and the frequency of correct productions versus errors. However, only the latter touched upon the crucial question of whether multiword sequences can yield errors when used incorrectly, and did so only informally.

In the present study, we systematically investigate the possibility that stored multiword sequences shape children's *wh*‐question non‐inversion errors. Take, for instance, the following correctly inverted and non‐inverted (errorful) forms (4‐5):

1. *What is she going to do ?*
* What she is going to do ? *

If strings that appear in the (potential) non‐inverted form, such as “*is going*,” and “*she is going*,” are highly frequent in the child's input, we might expect—given evidence that multiword sequences play a role in learning and processing—that the child will be more likely to produce the errorful form of this question. By the same token, we might expect the frequency of “*she going*” and “*is she going*” to alter this likelihood in the opposite direction. From this perspective, multiword sequences appearing in the correctly inverted and non‐inverted forms may be viewed as competing. This would be consistent with findings for individual words, where forms compete and high‐frequency items appear to “intrude,” leading to errorful productions (see Ambridge et al., 2015 for an overview of such findings).

In the present study, we therefore evaluate the role of multiword units in early *wh‐*question production by using distributional statistics from child‐directed speech to predict children's spontaneous errors of non‐inversion. We collect, from the entire English language portion of the CHILDES database (MacWhinney, 2000)^1^, occurrence statistics for words and higher‐order *n‐*grams, which are then used as predictors in logistic regression models of children's correctly inverted and errorful (uninverted) *wh‐* questions. This method allows us to evaluate the role played by multiword sequences identical to those that appear in the child's errorful, uninverted forms of questions while controlling for the statistics of sequences appearing in the correctly inverted forms, and vice‐versa.

## 2 METHODS

The corpus analysis can be divided into three distinct stages: (1) extraction of all child‐produced *wh‐* questions from a set of target corpora, followed by identification of uninversion errors; (2) collection of *n‐*gram statistics reflecting the ambient language environment; (3) mixed‐effects logistic regression modeling to determine which *n*‐gram statistics are predictive of uninversion errors in the extracted question set.

### 2.1 Corpus selection and preparation

We began by identifying, within the English portion of the CHILDES database (MacWhinney, 2000), the corpora with the greatest number of child *wh‐* questions. We used the top 12 such corpora rather than including the entire set of corpora in the database, in order to avoid additional noise arising from the large number of corpora with very few child *wh‐* questions (and thus little or nothing in the way of uninversion errors). Each of the 12 target corpora already fit our selection criteria of involving a single target child (rather than aggregating across multiple children) and spanning at least 1 year of development. The age range of each target child is provided in Table 1 along with citation information.

**TABLE 1:** Details of CHILDES corpora used in analysis of uninversion errors

Target Child	Corpus	Age Range
Abe	Kuczaj, 1977	2;04–5;00
Adam	Brown, 1973	2;03–5;02
Eleanor	Lieven et al., 2009	2;00–3;00
Ethan	Demuth & McCullough, 2009	0;11–2;11
Fraser	Lieven et al., 2009	2;00–3;01
Laura	Braunwald, 1976	1;05‐7;00
Lara	Rowland & Fletcher, 2006	1;09‐3;03
Lily	Demuth & McCullough, 2009	1;01‐4;00
Naima	Demuth & McCullough, 2009	0;11‐3;10
Ross	MacWhinney, 1991	1;04‐7;08
Sarah	Brown, 1973	2;03‐5;01
Thomas	Maslen et al., 2004	2;00‐4;11

Prior to analysis, each corpus was submitted to an automated procedure whereby codes, tags, and punctuation were removed, leaving only speaker identifiers and actual utterances. As an additional part of this procedure, contractions were split into their component words: for example, “*what's she doing*” was re‐coded as “*what is she doing*.” As corpus annotation differs in terms of how contractions are transcribed (leading to arbitrary noise), this step helped to standardize *n‐*gram frequencies for *wh‐* words and auxiliaries across all questions. As a final step, we collapsed the pronouns “*she*” and “*he*” into a single form to control for individual differences across children's exposure to gender pronouns.

### 2.2 *Wh‐* question and uninversion error candidate extraction and coding

For each of the 12 target corpora, child‐produced *wh‐* questions were automatically extracted by utilizing the standard default morphological tagging included in CHILDES. All extracted questions featured a *wh‐* word in the initial position and were followed immediately by an auxiliary. This yielded ≈13,000 child‐produced *wh*‐ questions across the 12 corpora.

In order to automatically identify potential uninversion errors, we also extracted all child‐produced questions featuring a *wh‐* word in the initial position but not immediately followed by an auxiliary. These candidate items were then manually coded for error type by the first author, yielding a total of 300 uninversion errors produced across the target children. *Wh*‐ questions featuring an error type other than uninversion (such as doubling [∼100; e.g., “Why can I can't eat the crisps?*”] or omission [∼5000; “What you doing out there?*”] errors) were excluded from the dataset. Analyses were restricted to non‐subject *wh‐* questions produced before the age of 5 years, given that only two of the corpora extended beyond this point in the target child's development. Finally, as discussed below, our analyses focused on the role of *n*‐grams up to the third order, including the first 5 unigrams, 4 bigrams, and 3 trigrams occurring at the beginning of each question (questions without at least 5 unigrams were excluded). The final resulting dataset consisted of 5499 questions, with an uninversion error rate of 4.4%.

Within this final dataset, there were individual differences in the rate of uninversion errors across the 12 children, ranging from 16% (Adam) to 0% (Lily and Ethan), with a range in between: 11% (Abe), 8% (Naima), 6% (Sarah), 4% (Laura), 3% (Fraser), 2% (Thomas and Ross), and 1% (Eleanor and Lara). We include child as a random factor in our analyses (see below).

### 2.3 *N*‐gram data collection

For every question that a child produced (whether they produced the correct or the uninverted form), we (1) generated both the correct and the uninverted form, then (2) collected the input *n‐*gram statistics for each. The first step was achieved as follows: For questions produced in uninverted form, we simply created a corresponding “correct” version by hand. For the far greater number which were produced in a correct form, we employed an automated procedure to generate the hypothetical, corresponding uninverted form. The second and third words could not simply be swapped because many questions featured multiword subject noun phrases, such as “*where is my red truck?*” Thus, to automatically achieve the appropriate uninverted form, we first shallow‐parsed utterances (Punyakanok & Roth, 2001). Shallow parsers function to segment out the non‐overlapping, non‐embedded phrases in a text. For instance, the shallow parser output for the previous example would be: “[where] [is] [my red ball].” After submitting correctly inverted questions to the shallow parser, we simply switched the second and third chunks, yielding the relevant, uninverted errorful forms, such as “*where my red ball is?*”

The second step was to calculate *n*‐gram statistics for both the correct and the uninverted forms of each question. With the aim of capturing statistics which accurately reflect the nature of child‐directed speech in English, we gathered *n‐*gram frequencies from the entire English (UK and US) portion of the CHILDES database. This allowed us to reduce potential issues of data sparseness arising from corpus size (e.g., Manning & Schütze, 1999). The resulting aggregated corpus was prepared for data collection following the same procedure described in the above subsection. Frequencies were collected for unigrams (single words), bigrams (word pairs), and trigrams (word triplets), which were then applied to each of the *wh‐* questions extracted for the 12 target child corpora. As an example, for the question “*what are you doing there*,” five unigram counts (one for each of five word positions: *“what,” “are,” “you,” “doing,”* and *“there”*), four bigram counts (one for each of three word pair positions: *“what are,” “are you,”* “*you doing,”* and *“doing there”*), and three trigram counts (one for each word triplet: “*what are you,” “are you doing,”* and *“you doing there”*) were calculated, with the frequencies themselves being derived from across all utterances in the aggregated corpus. For the same example question, the *n*‐gram frequencies for the corresponding uninverted form (*“what you are doing there?”*) were also calculated: four bigram counts (one for each word pair: *“what you,” “you are,”* etc.) and three trigram counts (one for each word triplet: *“what you are,”* etc.). Thus, the above procedures resulted in unigram, bigram, and trigram statistics for each position across all questions in their correct as well as uninverted forms. These *n*‐grams were all based on individual words; no words were bound together into compounds except where already existing as compounds in the corpus.^2^

### 2.4 Analysis

To evaluate the predictive relationship between multiword sequence frequency and uninversion errors, we used mixed‐effects logistic regression modeling (e.g., Agresti, 2002).^3^ We carried out a set of model comparisons to determine which *n‐*gram frequencies were uniquely predictive of uninversion errors. This involved selecting predictors at each *n‐*gram level separately using a leave‐one‐out procedure, starting at the unigram level before moving to the bigram level, followed by the trigram level. As we moved from one level to the next, any lower‐level predictors that were found to explain unique variance were carried over. Thus, for a higher‐order *n‐*gram (e.g., the trigram “*he can go”* from the errorful question “where he can go?*”) to reach significance, it would need to provide predictive value over and above that provided by individual words (e.g., the unigram *“can”*) or shorter sequences (e.g., the bigram *“can go”*). Thus, the model comparison procedure was designed to privilege lower‐order *n*‐grams in the selection process; this not only allowed us to provide a more conservative test of the hypothesized role for higher‐order *n*‐grams, but also offered greater transparency and interpretability, as it enables direct evaluation of the relative informativity of *n*‐grams at each level as well as overall. Moreover, this incremental procedure allowed us to sidestep issues presented by multicollinearity (which would logically be greatest between rather than within levels, since unigrams are nested in bigrams, and so on) in selecting predictors. The emphasis is on uncovering which variables, at each step, explain unique variance over and above the others.

To carry out the logistic regression analyses, questions originally produced by the target children in correctly inverted form were coded as 0, while questions produced in an errorful, uninverted form were coded as one. *N‐*gram frequencies were then used as predictors of this binary variable. Predictors were log‐transformed, mean‐centered and scaled. All model comparisons were carried out using likelihood ratio tests. All models included a random intercept for child, to reflect the fact that the 12 target children differed from one another in overall error rate, while by‐child random slopes were also included for each predictor, to reflect the fact that the 12 target children may differ in the extent to which their errors could be predicted by the various *n‐*gram frequencies. It was possible to include random slopes for all predictors (Barr et al., 2013). The incremental way in which first unigrams, then bigrams and then trigrams were considered for inclusion in our models meant that when unigrams were being considered, all unigram positions were included as random effects; when bigrams were being included, all unigrams that were found to explain significant variance as well as all bigrams were included as random effects, and so on.

Beginning at the unigram level, the full baseline model included fixed effects of the first five unigrams as well as random effects (by child). This was then compared to five subsequent models, each leaving out the fixed effect term for a different unigram. Where removal of a particular unigram frequency variable harmed model fit, according to likelihood ratio tests, that variable was held over for the next level of model comparisons, where the same procedure described for unigrams was then carried out for the first four bigrams. At this level, random (by child) and fixed effects for the surviving unigrams were included in each model alongside random and fixed effects for bigrams from both the correctly inverted and the corresponding errorful forms. Bigrams (from either the correctly inverted or errorful, non‐inverted forms of each question) which harmed model fit to a statistically significant effect by their removal were then retained for the final set of model comparisons. Thus, in addition to the surviving unigrams from the previous step, surviving bigrams (which could be from either the correctly‐inverted question forms, or the errorful, non‐inverted forms), were held over for the final set of model comparisons, which took place at the trigram level. For this final set of comparisons, the same procedure was followed once more (with random and fixed effects for the held‐over unigrams and bigrams included).^4^

## 3 RESULTS

**FIGURE 1:**
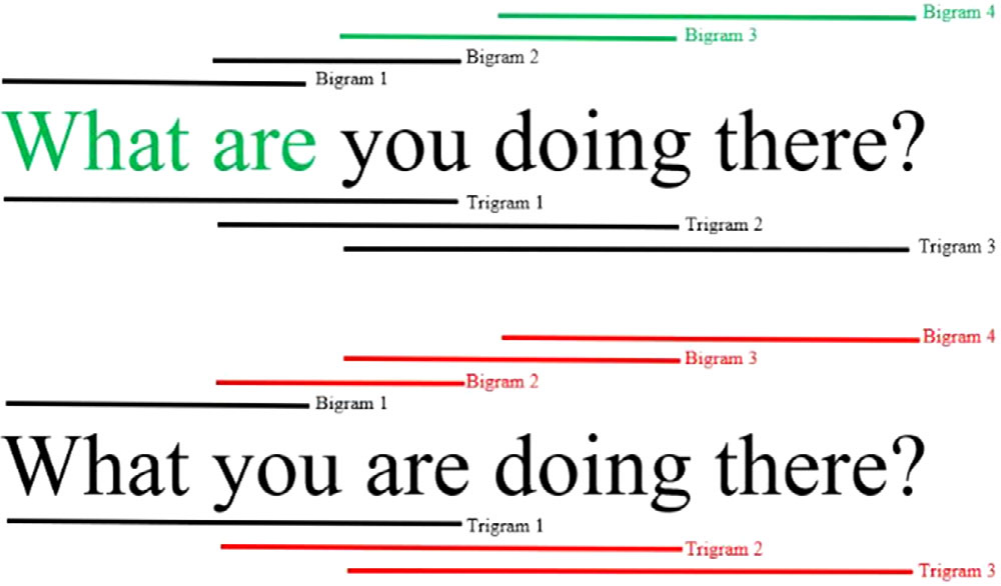
Unigrams (individual words), bigrams, and trigrams for the correctly inverted (top) and corresponding errorful (bottom) forms of the example question *What are you doing there?* N‐grams excluded from the final statistical model are shown in black. N‐grams retained in the final statistical model are shown as green/red words (unigrams) and green/red line (bigrams and trigrams). Note that this figure mixes the example level with the general design level for illustration purposes

The model comparison procedure (described above) yielded nine separate *n‐*gram predictors (see Figure 1). Using the question “*what are you doing there?”* as an example, these included: The first two unigrams (*what* and *are*) and third and fourth bigrams from the correctly inverted question forms (*you doing* and *doing there*); and the second (*you are*), third (*are doing*), and fourth (*doing there*) bigrams as well as the second (*you are doing*) and third (*are doing there*) trigrams from the errorful (uninverted) question forms.^5^

The log‐likelihood, chi‐squared value, and *p‐*value for each model comparison is shown in Table 2, alongside example *n*‐grams.

**TABLE 2:** Results of model comparisons

Left‐out Predictor	Log‐likelihood	*χ^2^*	*p*‐value	Ex.
Unigram (full/baseline)	−702.13	–	–	–
Unigram 1	−705.6	6.95	0.00 **	*what*
Unigram 2	−707.16	10.07	0.00 **	*are*
Unigram 3	−702.27	0.29	0.59	*you*
Unigram 4	−702.13	0.00	0.97	*doing*
Unigram 5	−702.20	0.14	0.71	*there*
Bigram (full/baseline)	−626.40	–	–	–
Bigram 1	−627.28	1.76	0.19	*what are*
Bigram 2	−627.20	1.59	0.21	*are you*
Bigram 3	−631.41	10.01	0.00 **	*you doing*
Bigram 4	−632.68	12.55	0.00 ***	*doing there*
Trigram (full/baseline)	−614.62	–	–	–
Trigram 1	−615.44	1.641	0.2002	*what are you*
Trigram 2	−615.69	2.141	0.1434	*are you doing*
Trigram 3	−614.67	0.103	0.748	*you doing there*
Uninverted Bigram (full/baseline)	−626.40	–	–	–
Uninverted Bigram 1	−626.42	0.02	0.88	*what you*
Uninverted Bigram 2	−634.79	16.77	0.00 ***	*you are*
Uninverted Bigram 3	−634.87	16.94	0.00 ***	*are doing*
Uninverted Bigram 4	−632.5	12.19	0.00 ***	*doing there*
Uninverted Trigram (full/baseline)	−614.62	–	–	–
Uninverted Trigram 1	−614.87	0.505	0.4772	*what you are*
Uninverted Trigram 2	−617.55	5.874	0.02 *	*you are doing*
Uninverted Trigram 3	−618.41	7.582	0.01 **	*are doing there*

*Note*. Errorful (uninverted) questions are coded as 1, while correctly inverted questions are coded as 0.

We report fixed effect estimates for the final model in Table 3. As can be seen, the first and second unigram frequencies (corresponding to the *wh‐* word and auxiliary, e.g., *what* and *are*, in the example question *what are you doing there?*) had negative estimates, indicating lower likelihood of an uninversion error with more frequent items. The same held for the third and fourth bigram frequencies (e.g., *you doing* and *doing there*). Importantly, for *n*‐gram predictors drawn from the errorful, uninverted question forms, the estimate was positive. This means that the higher the *n‐*gram frequency for the uninverted form of a question, the more likely it was for that question to have been produced in its uninverted form (see Table 3 for further examples).^6^

**TABLE 3:** Results of full model

Item	β	Std. Error	Ex.
Intercept	−4.24	0.34	*–*
Uni 1	−0.69	0.24	*what*
Uni 2	−0.78	0.12	*Are*
Bi 3	−0.73	0.14	*you doing*
Bi 4	−0.95	0.20	*doing there*
Bi 2 (*uninv*.)	0.67	0.15	*you are*
Bi 3 (*uninv*.)	0.59	0.15	*are doing*
Bi 4 (*uninv*.)	0.34	0.16	*doing there*
Tri 2 (*uninv*.)	0.10	0.15	*you are doing*
Tri 3 (*uninv*.)	0.56	0.16	*are doing there*

*Note*. Errorful (uninverted) questions are coded as 1, while correctly inverted questions are coded as 0. Beta coefficients are included for transparency; conclusions regarding the significance of variables are based, instead, on the model comparisons (described above).

## 4 DISCUSSION

The present study represents, to our knowledge, the most rigorous treatment of input frequencies in an analysis of question errors to date. We find that corpus frequencies for *n*‐gram sequences appearing in the correctly‐formed, “target” question are predictive of lower uninversion rates, while *n*‐gram frequencies from the non‐inverted form predict higher uninversion rates. This finding is consistent with previous evidence that children actively draw upon stored, multiword units (e.g., *“go to the store”*) during on‐line language processing (e.g., Arnon & Clark, 2011; Bannard & Matthews, 2008). Consider, as an example, our finding that non‐inverted trigrams are predictive of non‐inversion errors such as “*Where we can go today?*” The more strongly a sequence like *”we can go”* holds together as a unit for an individual child, the less likely the child may be to disrupt that sequence by fronting the auxiliary *can*. This general notion is consistent with findings that frequent items protect against error across a number of linguistic domains (cf. Ambridge et al., 2015), as well as findings that errors can be caused by the intrusion of overlearned sequences across all kinds of human action (e.g., Bannard et al., 2019).

Thus, in addition to supporting the proposal that material learned from declarative utterances can drive systematic errors, our findings weigh in favor of previous proposals that children rely on lexically‐based representations in question formation (e.g., Rowland & Pine, 2000). Previous work has argued for the importance of *wh‐ + auxiliary* combinations as units. In our model, this combination did not prove to be among the selected variables. Instead, the frequencies of the *wh‐* word and the *auxiliary* were included as separate entities. This is due to the collinearity between the component words and their combination: the hierarchical, iterative way in which we determined the significance of predictors meant that the lower‐order unigrams were selected at the expense of the higher. While this does not contradict the finding that the *wh+aux* combination has predictive value, it does indicate that the combinations may not have unique explanatory value over their component words. We instead found that other multiword sequences—those later in the question and those found in the uninverted form, and thus not considered in prior work—were significant predictors of non‐inversion error. We therefore consider our findings to be consistent with the spirit if not the letter of prior usage‐based work.

Importantly, our findings are also consistent with a number of theoretical proposals which do *not* assume holistic storage of multiword units (cf. contributions in Wiechmann et al., 2013). Our interpretation depends only on the idea that children have mental representations that derive from repeated exposure to particular word sequences: whether or not those sequences are stored as concrete units, the key notion we are arguing for is that a child's competence with such a sequence (and, therefore, the role of such a sequence in question production) cannot be explained *solely* by experience with the component parts, but depends also on prior experience with the entire string. Such a view is compatible with, e.g., connectionist approaches, or those based on discriminative learning (e.g., Baayen et al., 2013).

The present study offers an important additional line of evidence supporting usage‐based approaches, especially accounts of language development which stress the importance of multiword chunks (e.g., McCauley & Christiansen, 2019a; Theakston & Lieven, 2017), including exemplar‐based approaches (Ambridge, 2019). Accounts of *wh‐* question development rooted in theoretical models based solely on structural considerations, or which eschew the notion of lexically‐based representations in early development, may not be able to accommodate these findings so straightforwardly (e.g., de Villiers, 1991). Moreover, our findings make clear that any complete model of language production must consider distributional statistics in the broadest sense: rather than merely considering frequencies tied to the context or construct of interest (e.g., studying *wh‐* question formation by looking only at frequencies for items occurring in *wh*‐ questions themselves, such as *wh‐* word + auxiliary combinations), researchers must recognize that the frequency of word sequences encountered across the input can play a role.

## CONFLICT OF INTEREST

The authors declare no potential conflicts of interest.

## Data Availability

This study involved the analysis of a publicly available dataset. A URL for analysis code is provided in the main text.

## Footnotes

1 CHILDES data downloaded January 2017.

2 Minimum, mean, and maximum frequency counts for each n‐gram position is given for the analyzed questions are included in Appendix A.

3 While non‐inversion errors made up only 4.4% of the final dataset, this proportion was large enough, and with a large enough *n*, that our situation would be considered low‐risk for problems arising from asymmetry according to previous work on logistic regression modeling involving rare events data (cf. King & Zeng, 2001). Importantly, our analyses are not concerned with estimated odds but, rather, with whether individual predictors explain unique variance.

4 Dataframe and code may be accessed at https://osf.io/6t8fb/?view_only=f3b06308e14042cca9047638e94fd067

5 Owing to previous research raising the possibility that questions featuring auxiliary DO may be qualitatively different (e.g., Santelmann et al., 2002; Stromswold, 1990), we carried out a second set of analyses in which all questions featuring DO‐support were excluded from the dataset. All effects for *n*‐grams emerging from the leave‐one‐out procedure were retained in this version, with the exception of the second uninverted trigram, the exclusion of which lead only to a marginal decrease in model fit (χ = 3.4, *p *= 0.065).

6 In order to ensure that repeated questions (both within and across children) did not bias our results, we re‐ran the entire set of analyses after randomly excluding all but one instance of questions that occurred more than once in the dataset. Approximately 86% of the questions in the final dataset were unique, with most repeated items being correctly inverted. The most frequent errorful question (“*what I am going to do?”*) occurred only three times. The resulting model comparisons lead to the exact same *n*‐grams surviving the leave‐one‐out procedure as reported for the full dataset.

## APPENDIX A

### A.1

**TABLE A1:** CHILDES (English) frequency counts for N‐grams across dataset for all caregiver and child utterances

*N*‐gram	Caregiver Min. Freq	Caregiver Mean Freq.	Caregiver Max. Freq	Child Min Freq.	Child Mean Freq.	Child Max Freq.
**Unigram 1**	20,153	98,343.0391	219,514	7,414	30,833.4	50,444
**Unigram 2**	1,110	153,861.9	266,567	128	51,689.5	85,760
**Unigram 3**	0	241,017.3	508,191	1	68,353.7	187,512
**Unigram 4**	0	35,680.99	508,191	1	16,613.1	187,512
**Unigram 5**	0	71,296.2	508,191	1	25,878.5	187,512
**Bigram 1**	0	18,830.97	66,309	1	8,090.5	20,921
**Bigram 2**	0	16,062.03	66,796	1	2,978.7	7,216
**Bigram 3**	0	2,227.369	41,221	1	710.1	19,255
**Bigram 4**	0	2,622.009	66,796	1	737.4	14,432
**Trigram 1**	0	2,697.657	13,351	1	915.9	3,867
**Trigram 2**	0	609.1358	16,887	1	68.7	1,649
**Trigram 3**	0	265.8017	14,145	1	42.9	4,784
**Bigram 1 (Uninverted)**	0	1,348.902	6,884	1	669.9	20,921
**Bigram 2 (Uninverted)**	0	4,351.922	41,221	1	1,271.8	14,432
**Bigram 3 (Uninverted)**	0	593.7	43,418	1	336.4	9,373
**Bigram 4 (Uninverted)**	0	2762.7	66,796	1	780.8	20,921
**Trigram 1 (Uninverted)**	0	55.3	653	1	16.4	205
**Trigram 2 (Uninverted)**	0	84.9	3,540	1	24.3	1,977
**Trigram 3 (Uninverted)**	0	68.0	2,592	1	16.6	1,146
